# Net Benefits: A Multicountry Analysis of Observational Data Examining Associations between Insecticide-Treated Mosquito Nets and Health Outcomes

**DOI:** 10.1371/journal.pmed.1001091

**Published:** 2011-09-06

**Authors:** Stephen S. Lim, Nancy Fullman, Andrew Stokes, Nirmala Ravishankar, Felix Masiye, Christopher J. L. Murray, Emmanuela Gakidou

**Affiliations:** 1 Institute for Health Metrics and Evaluation, University of Washington, Seattle, Washington, United States of America; 2 Population Studies Center, University of Pennsylvania, Philadelphia, Pennsylvania, United States of America; 3 Abt Associates Inc., Bethesda, Maryland, United States of America; 4 Department of Economics, University of Zambia, Lusaka, Zambia; Kenya Medical Research Institute - Wellcome Trust Research Programme, Kenya

## Abstract

**Background:**

Several sub-Saharan African countries have rapidly scaled up the number of households that own insecticide-treated mosquito nets (ITNs). Although the efficacy of ITNs in trials has been shown, evidence on their impact under routine conditions is limited to a few countries and the extent to which the scale-up of ITNs has improved population health remains uncertain.

**Methods and Findings:**

We used matched logistic regression to assess the individual-level association between household ITN ownership or use in children under 5 years of age and the prevalence of parasitemia among children using six malaria indicator surveys (MIS) and one demographic and health survey. We used Cox proportional hazards models to assess the relationship between ITN household ownership and child mortality using 29 demographic and health surveys. The pooled relative reduction in parasitemia prevalence from random effects meta-analysis associated with household ownership of at least one ITN was 20% (95% confidence interval [CI] 3%–35%; *I* ^2^ = 73.5%, *p*<0.01 for *I* ^2^ value). Sleeping under an ITN was associated with a pooled relative reduction in parasitemia prevalence in children of 24% (95% CI 1%–42%; *I* ^2^ = 79.5%, *p*<0.001 for *I* ^2^ value). Ownership of at least one ITN was associated with a pooled relative reduction in mortality between 1 month and 5 years of age of 23% (95% CI 13–31%; *I* ^2^ = 25.6%, *p*>0.05 for *I* ^2^ value).

**Conclusions:**

Our findings across a number of sub-Saharan African countries were highly consistent with results from previous clinical trials. These findings suggest that the recent scale-up in ITN coverage has likely been accompanied by significant reductions in child mortality and that additional health gains could be achieved with further increases in ITN coverage in populations at risk of malaria.

*Please see later in the article for the Editors' Summary*

## Abstract 2

Stephen Lim and colleagues report findings from a multi-country analysis of household survey data on the association between possession of insecticide-treated mosquito nets and child mortality and parasitemia. Scale-up of net coverage was associated with a substantial reduction in childhood mortality and in parasitemia prevalence.

## Editors' Summary

**Background:**

Malaria is a major public health problem. Half the world's population is at risk of this parasitic disease, which kills a million people (mainly children living in sub-Saharan Africa) every year. Malaria is transmitted to people through the bites of infected night-flying mosquitoes. Soon after entering the human body, the parasite begins to replicate in red blood cells, bursting out every 2–3 days and infecting more red blood cells. The presence of the parasite in the bloodstream (parasitemia) causes malaria's characteristic fever and can cause fatal organ damage. Malaria can be prevented by controlling the mosquitoes that spread the parasite and by owning and sleeping under insecticide-treated nets (ITNs) to avoid mosquito bites. In trials, ITN use reduced parasitemia in young children by about 13% and deaths among children by about 18%. Consequently, the widespread provision of ITNs is a mainstay of the World Health Organization's efforts to control malaria, and in 2005 the World Health Assembly agreed a target of providing ITNs for 80% of the people at risk of malaria by 2010.

**Why Was This Study Done?:**

Although progress towards this goal has been variable, several sub-Saharan African countries have rapidly scaled up the fraction of households that own ITNs from near zero to more than 60% with the support of international donors. But has this scale-up of ITN coverage been accompanied by improvements in health outcomes similar to those seen in the trials of ITNs? ITNs may not work as well under routine conditions as in trials because of, for example, the use of nets that are no longer impregnated with active insecticide; nets have to be retreated regularly with insecticide to maintain their protection against mosquitoes. Unfortunately, in many countries in sub-Saharan Africa, health information systems are weak and incomplete records of deaths are kept, which makes it impossible to determine the rates of malaria-specific morbidity (illness) and mortality (deaths) accurately. In this study, the researchers use data collected in household surveys to examine the association between ITN ownership in a number of sub-Saharan African countries and two specific outcomes—the proportion of the population with parasitemia, and child mortality.

**What Did the Researchers Do and Find?:**

The researchers used a statistical method to assess the association between household ITN ownership or use in young children and the prevalence of parasitemia among children using data from a set of household surveys. They looked specifically at the relationship between ITN household ownership and child mortality using data from 29 surveys undertaken in 22 sub-Saharan African countries. They then pooled the results of the individual surveys. The pooled relative reduction in parasitemia prevalence among children associated with household ownership of at least one ITN was 20%. That is, averaged out over the countries studied, household ITN ownership was associated with a reduction of around a fifth in the prevalence of parasitemia. The pooled relative reduction of parasitemia prevalence associated with children sleeping under an ITN was 24%. Finally, the pooled relative reduction in mortality between 1 month and 5 years old associated with household ITN ownership was 23%.

**What Do These Findings Mean?:**

These findings suggest that the rapid scale-up in ITN coverage that has occurred in several sub-Saharan African countries has been accompanied by significant reductions in child deaths. Importantly, these findings are highly consistent with those from trials of ITNs. The accuracy of these findings may be affected by some aspects of the study design. For example, because the study uses observational data, it is possible that people who own ITNs share other characteristics that are actually responsible for the reduction in parasitemia prevalence and childhood deaths. Nevertheless, these findings add to the body of evidence that ITNs are effective in routine use. Thus, they support continued efforts to scale-up ITN coverage in sub-Saharan Africa and highlight the importance of maintaining ITN coverage in countries that have already successfully scaled up coverage.

**Additional Information:**

Please access these Web sites via the online version of this summary at http://dx.doi.org/10.1371/journal.pmed.1001091.

- The Institute for Health Metrics and Evaluation provides visualizations and datasets for a range of global health indicators including child mortality and insecticide treated bed net coverage
- Information is available from the World Health Organization on malaria (in several languages); the 2010 World Malaria Report provides details of the current global malaria situation
- The US Centers for Disease Control and Prevention provide information on malaria and on insecticide-treated bed nets
- The Roll Back Malaria Partnership provides information on the global control of malaria, malaria in Africa and insecticide-treated bed nets, and access to Malaria Indicator Survey datasets
- Information is also available about the Demographic and Health Surveys
- MedlinePlus provides links to additional information on malaria (in English and Spanish)

## Introduction

Several sub-Saharan African countries, with support from international donors, have rapidly scaled up the fraction of households that own insecticide-treated mosquito nets (ITNs) from essentially zero to above 60% over the last decade [1]. Although there has been variable progress across countries, the push to increase ITN coverage continues with more dramatic improvements seen in the last few years [2].

The large expansion in the distribution of ITNs has been motivated by evidence from cluster-randomized controlled trials (RCTs) that showed pooled relative reductions in child mortality of 18% [3] and parasite prevalence of 13% as a result of net use [4]. There are several reasons why improvements in health outcomes of the same magnitude might not be observed under routine conditions [5]. These include, for example, reduced net integrity and improper use. As a result, efforts should be made to measure not only the coverage of ITNs, but also their impact on health outcomes under real-world settings [6],[7].

Evaluating the impact of malaria control strategies, including the scale-up of ITNs on health outcomes, is difficult. Weak routine health information and vital registration systems mean that it is often not possible to accurately determine malaria-specific mortality and morbidity. Evidence about the impact of ITNs under routine conditions has been limited to selected studies such as those conducted in rural Kenya [8], the Gambia [9], Tanzania [10],[11], and rural Somalia [12]. These studies, however, have used different approaches to assess the relationship between ITNs and health outcomes and represent only some of the countries where ITN coverage has been scaled up.

In this paper, using routinely collected household surveys, we demonstrate an approach to measure in a comparable way the association between use and ownership of ITNs and parasitemia prevalence and child mortality across a large number of countries where ITNs have been distributed. This method quantifies the impact of ITNs, under routine conditions, to allow a better understanding of the effect on child health of the recent ITN scale-up.

## Methods

### Data

We considered all demographic and health surveys (DHS) and malaria indicator surveys (MIS) from sub-Saharan Africa countries conducted since 2000 for which the unit-record data were available. Prior to 2000, ITN ownership and use in sub-Saharan Africa was universally low [13]. We included only surveys that collected data on the health outcomes of interest (child mortality or parasitemia prevalence) as well as information on ITN ownership and use (including when the ITN was received or purchased, and when it was retreated) and all covariates specified in the analyses. We excluded the Ghana DHS conducted in 2003 as no child deaths were observed in the small number of households that owned ITNs. The results on the association between ITNs and child mortality are based on 29 DHS in 22 sub-Saharan African countries, while the results on the association between ITNs and parasitemia prevalence are based on 6 MIS and one DHS from seven sub-Saharan African countries.

### Ownership and Use of ITNs

Mosquito nets were classified as conventional ITNs, which require retreatment at least every year, or long-lasting insecticide-treated mosquito nets (LLINs), which should be replaced after 3 y [14]. While the data collection procedure varied slightly across surveys, in general survey interviewers visually confirmed presence of nets in the household and recorded the following information for each net in a net roster: how long ago it was acquired; brand, specifically, if it is an LLIN; and for conventional ITNs, how long ago the net was last treated. We considered a net to be an ITN if it was an LLIN that was less than or equal to 3 y old or a conventional ITN that was less than or equal to 1 y old or had been retreated in the last year. The net roster was linked to the household roster and this was used to identify which member slept under the net the previous night.

Using this information, we estimated three variables of net ownership and use, two at the household level and one at the child (aged less than 5 y) level: (i) whether or not the household owned an ITN, (ii) how many ITNs each household owned per household member, and (iii) whether the child slept under an ITN the night prior to the survey.

### Health Outcomes

Parasitemia in children under the age of 5 y of age was ascertained in surveys using a rapid diagnostic test (RDT) and/or microscopy using thick or thin blood smears. Survey data and documentation did not always indicate whether the positive result was determined from RDT or microscopy.

Survival of children from age 1 mo to 59 mo was determined from complete birth histories of women of reproductive age (15 to 49 y). We examined mortality between age 1 mo and 59 mo as this is the same age period used in RCTs and previous observational studies [4],[8]; malaria deaths in the neonatal period are very rare.

### Malaria Transmission Intensity

All analyses controlled for the effect of malaria transmission intensity. To determine malaria transmission intensity, we used global positioning system (GPS) coordinates for each of the primary sampling units (PSUs) in the MIS or DHS and linked this to data on malaria transmission from the Malaria Atlas Project (http://www.map.ox.ac.uk; [15]) using ArcGIS. All households in the PSU were assigned the malaria transmission based on the PSU-level GPS coordinates. We categorized malaria transmission intensity into the following categories: (i) high transmission, defined as *Pf*PR_2–10_ or *P. falciparum* parasite rate (2 to 10 y) between 40%–100%; (ii) medium transmission, defined as *Pf*PR_2–10_ between 5%–40%; and (iii) low transmission, defined as *Pf*PR_2–10_ between 0%–5% [16]. Seven DHS did not have PSU-level GPS coordinates available (Benin 2006, Congo 2005, Eritrea 2002, Niger 2006, Rwanda 2000, São Tomé & Príncipe 2008, and Zambia 2001–2002). For these seven surveys, households were assigned a malaria transmission category on the basis of the average population-weighted parasite rate in the province where the household was located.

### Effect of ITN Ownership and Use in Children under 5 on Parasitemia Prevalence

We examined the effect of ITN ownership and use on parasitemia prevalence using exact matching. The literature on the use of matching for causal inferences is sophisticated and growing, and includes several applications in global health and evaluations of health policies [17]–[21]. Matching provides a way of preprocessing the data so that the treated group is as similar to the control group as possible, thus making the treatment variable (in this case, ITN ownership or ITN use) as independent of the background characteristics as possible. By breaking or reducing the link between the treatment variable and the control variables, matching makes estimates based on subsequent analyses less dependent on model specification.

Within each survey, we exactly matched children who live in a household that owns an ITN or children who slept under an ITN the night prior to the survey to children from households without an ITN on the basis of the following covariates: (i) age of the child (0–1, 2–3, 4+ y); (ii) mother's education (none, any); (ii) urban/rural residence; and (iv) malaria transmission intensity. We implemented the exact matching procedure using the MatchIt software in R [22].

We then used logistic regression on the matched dataset to provide added control of potential confounders using the following covariates: (i) age of the child (0–1, 2–3, 4+ y); (ii) mother's education (none, primary, secondary or more); (iii) urban/rural residence; (iv) household wealth quintile; (v) malaria transmission intensity category; and (vi) wet or dry season at the time of the survey. We estimated household wealth using information on asset ownership [23]–[25].

A separate analysis was conducted for each survey and we determined the odds ratio (OR) associated with ITN ownership or use. We determined a pooled OR across all surveys using DerSimonian-Laird random effects meta-analysis [26].

### Effect of ITN Ownership on Child Mortality

We used complete birth history data from DHS to construct a retrospective cohort that traces survival of children from age 1 mo to 59 mo for the 3 y prior to the survey. From the household net roster, using the information on when each net was acquired and/or retreated, we determined household ownership of an ITN for each month during the 3 y prior to the survey. As the surveys only record use of ITNs for children who are alive at the time of the survey, we were not able to study the relationship between ITN use and child mortality.

We analyzed the relationship between household ownership of ITNs and child mortality using Cox proportional hazards models where analysis time was the age of the child in months. We controlled for the following covariates: (i) maternal age (in 5-y age groups); (ii) parity and birth interval (less than 12 mo, 12–23 mo, greater or equal to 24 mo or first born); (iii) sex of the child; (iv) single or multiple birth; (v) maternal education (no education, less than primary, less than secondary, secondary or more); (vi) household wealth quintile; (vii) urban/rural residence; (viii) skilled birth attendance (SBA) coverage at the PSU level; (ix) three-dose diphtheria, pertussis and tetanus (DPT3) immunization coverage at the PSU-level; (x) calendar year; (xi) malaria transmission intensity; and (xii) wet or dry season specific to the month of the observation. Wet and dry seasons were determined from the Mapping Malaria Risk in Africa project (http://www.mara.org.za/).

A separate analysis was conducted for each survey and we determined the relative risk (RR) of child mortality associated with ITN ownership. We determined a pooled RR across all surveys using DerSimonian-Laird random effects meta-analysis [26]. We examined the sensitivity of the results to recall bias by restricting the analysis to observations for just the one year prior to the survey.

### Effect of ITNs by Malaria Transmission Intensity, Number of ITNs Owned, and Urban and Rural Residence

Malaria transmission varies considerably within countries and it is likely that the effect of ITNs varies by transmission level. The effect of ITN ownership may also vary according to the number of ITNs owned by the household. Finally, the majority of RCTs and observational studies of ITNs were conducted in rural areas and the effect of ITNs in urban areas is less well characterized.

To test for these effects, we pooled individual observations from all surveys and grouped observations by transmission intensity (high, medium, and low), the number of ITNs owned per household member (0, <0.25 ITNs per household member, ≥0.25 ITNs per household member), and urban or rural residence. We ran separate models for each stratum. For the analysis of child mortality, we included a random effect term across surveys to capture systematic variation in the outcome across surveys. We did not include this term for parasitemia prevalence given the small number of surveys included.

All analyses were conducted in Stata 11 (Stata Corporation) and R 2.9.2 (University of Auckland).

## Results

Table 1 describes the characteristics of the 29 surveys included in the analysis of child mortality; Table 2 provides information about the seven surveys included in the analysis of parasitemia prevalence. These surveys cover the majority of malaria-endemic countries from sub-Saharan Africa with varying sized populations at risk of malaria. ITN household ownership coverage at the time of the survey ranged from less than 2% to almost 60% of households.

**Table 1:** Characteristics of surveys included in the analysis of child mortality.

Country	Survey	Year of Survey	*n* Households	Percent Households in Transmission Area			Household ITN Ownership (%)	Survival Analysis	
				High	Medium	Low		*n* Months of Observation	*n* Deaths
**Benin**	DHS	2001	5,769	86.4	13.0	0.6	3.7	143,784	274
**Benin**	DHS	2006	17,511	100.0	0.0	0.0	35.3	478,667	691
**Burkina Faso**	DHS	2003	9,097	99.0	1.0	0.0	9.5	289,592	681
**Cameroon**	DHS	2004	10,462	71.3	28.4	0.3	5.8	18,635	32
**Congo**	DHS	2005	5,879	100.0	0.0	0.0	8.0	133,967	185
**DRC**	DHS	2007	8,886	68.4	27.1	4.5	11.5	252,993	474
**Eritrea**	DHS	2002	9,824	0.0	64.7	35.3	3.6	226,380	168
**Ethiopia**	DHS	2005	13,721	0.0	38.7	61.3	3.5	306,171	355
**Ghana**	DHS	2008	11,778	79.3	16.0	4.7	39.5	90,434	75
**Kenya**	DHS	2003	8,561	3.1	33.6	64.3	8.9	175,527	236
**Madagascar**	DHS	2003–2004	4,223	18.3	81.7	0.0	2.2	153,711	124
**Madagascar**	DHS	2008–2009	17,857	18.5	73.1	8.4	42.9	345,757	238
**Malawi**	DHS	2004–2005	13,664	57.0	42.8	0.2	27.6	302,931	487
**Mali**	DHS	2006	12,998	70.4	25.2	4.4	47.1	403,769	892
**Namibia**	DHS	2006–2007	9,200	0.0	51.5	48.5	15.6	153,299	137
**Niger**	DHS	2006	7,660	0.0	90.6	9.4	50.3	274,681	608
**Nigeria**	DHS	2003	7,225	80.9	19.1	0.0	1.9	166,309	427
**Nigeria**	DHS	2008	34,070	79.8	20.0	0.2	10.7	833,164	1,659
**Rwanda**	DHS	2000	9,696	0.0	88.0	12.0	1.5	325,703	547
**Rwanda**	DHS	2005	10,272	3.5	52.6	43.9	15.9	248,787	425
**Rwanda**	DHS	2007–2008	7,377	1.6	58.2	40.2	50.5	154,988	143
**STP**	DHS	2008–2009	3,536	0.0	100.0	0.0	34.8	47,810	20
**Sierra Leone**	DHS	2008	7,284	83.3	13.5	3.2	32.8	163,408	264
**Tanzania**	DHS	2004–2005	9,735	16.4	57.0	26.6	25.9	245,829	301
**Uganda**	DHS	2006	8,870	30.6	65.7	3.7	19.1	240,116	385
**Zambia**	DHS	2001–2002	7,126	16.1	83.9	0.0	12.4	298,866	517
**Zambia**	DHS	2007	7,164	0.2	99.8	0.0	54.2	184,023	251
**Zimbabwe**	DHS	2005–2006	9,285	0.0	22.0	78.0	6.9	162,615	171

DRC, Democratic Republic of Congo; STP, São Tomé & Príncipe.

**Table 2:** Characteristics of surveys included in the analysis of parasitemia prevalence.

Country	Survey	Year of Survey	*n* House-holds	Percent Households in Transmission Area			ITN Coverage Indicators (%)		Child Parasitemia Measurements	
				High	Medium	Low	Ownership	Use	Total	*n* Positive
**Angola**	MIS	2006–2007	2,599	45.0	53.0	2.0	26.6	17.7	1,263	276
**Liberia**	MIS	2008–2009	4,162	91.2	0.0	8.8	41.7	26.4	1,296	419
**Rwanda**	DHS	2007–2008	7,377	1.6	58.2	40.2	50.5	55.7	2,509	67
**Senegal**	MIS	2008–2009	9,291	0.0	97.3	2.7	57.5	23.0	3,702	238
**Tanzania**	MIS	2007–2008	8,497	23.3	42.3	34.4	44.5	25.7	5,680	712
**Uganda**	MIS	2009–2010	4,421	37.0	59.8	3.2	42.5	32.8	2,108	852
**Zambia**	MIS	2006	2,999	21.5	78.0	0.5	44.4	22.8	947^a^	203^a^

a Parasitemia measurements were available for 1,817 children with 380 testing positive, but only 947 children's slide data could be properly linked to their respective household's bednet information.

**Figure 1:**
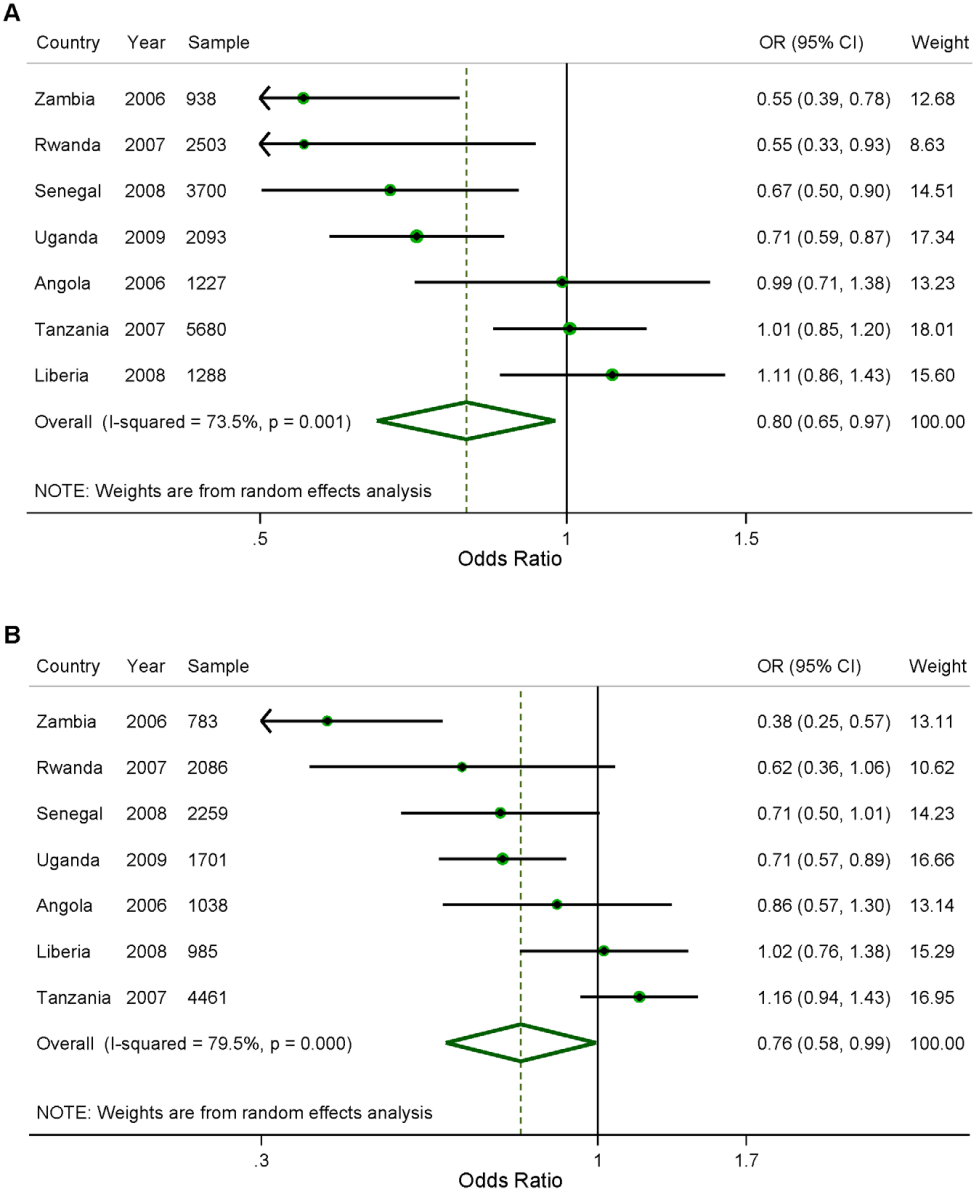
Effect of (A) ITN household ownership; and (B) ITN use in children under five, on prevalence of parasitemia.

Figure 1A shows the results of the analysis of the effect of household ownership of at least one ITN on the prevalence of parasitemia. Four countries demonstrated a statistically significant association between ITN ownership and parasitemia prevalence: Zambia with a 45% relative reduction in parasitemia prevalence (95% confidence interval [CI] 22%–61%); Rwanda with a 45% relative reduction (95% CI 7%–67%); Senegal with a 33% relative reduction (95% CI 10%–50%); and Uganda with a 29% relative reduction (95% CI, 13%–41%). Across the seven surveys, there was a significant pooled reduction in parasitemia prevalence of 20% (95% CI 3%–35%) associated with household ownership of an ITN. There was, however, significant heterogeneity in the association between ITN household ownership and parasitemia prevalence (*I* ^2^ = 73.5%, *p*<0.01). The pooled effect on the prevalence of parasitemia of children sleeping under an ITN the previous night (Figure 1B) was of a similar magnitude (relative reduction of 24%, 95% CI 1%–42%; *I* ^2^ = 79.5%, *p*<0.001) and not significantly different from the pooled effect on parasitemia of ITN ownership (*p*>0.05).

**Figure 2:**
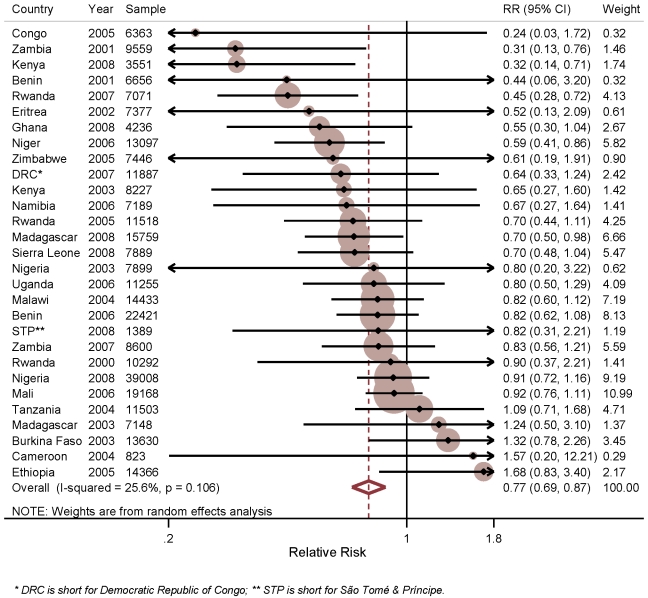
Effect of ITN household ownership on all-cause mortality among children 1 mo to 59 mo of age.

Figure 2 shows the results of the analysis of the effect of household ownership of at least one ITN on child mortality. In the individual surveys, there were statistically significant reductions in five surveys: Zambia 2001–2002 with a 69% RR reduction in child mortality (95% CI 24%–87%); Kenya 2008–2009 with a 68% RR reduction (95% CI 29%–86%); Rwanda 2007–2008 with a 55% RR reduction (95% CI 28%–72%); Niger 2006 with a 41% RR reduction (95% CI 14%–59%); and Madagascar 2008 with a 30% RR reduction (95% CI 2%–50%). Across the 29 surveys, there was a statistically significant pooled RR reduction in child mortality of 23% (95% CI 13%–31%) with the effect being consistent across the 29 surveys (*I* ^2^ = 25.6%, *p*>0.05 for the *I* ^2^ value). Restricting the analysis of ITN ownership on child mortality to observations in the 1 y prior to the survey, and thereby reducing the influence of recall bias, did not markedly change the estimated mean effect of ITN ownership (unpublished data).

Tables 3 and 4 show results of the logistic regression of ITN household ownership and use in children under-five on parasitemia by malaria transmission risk. The effect of ITN household ownership and use in children under-five were statistically the same across the three levels of transmission risk (*p*>0.05). In general, wet season, increasing child age, lower maternal education, and lower household wealth were significantly associated with higher odds of parasitemia (Tables 3 and 4). Table 5 shows the result of the Cox Proportional Hazards model of ITN household ownership on child mortality by transmission level. There were no statistically significant differences in the effect of ITNs on child mortality by malaria transmission level (*p*>0.05). In general, wet season, shorter birth intervals, a multiple birth, older maternal age, lower maternal education, lower household wealth, fewer household members, lower coverage of other childhood immunization, and skilled birth attendance were associated with higher probability of child mortality (Table 5). All the relationships observed between child mortality and parasitemia and the covariates controlled for are as expected and support the validity of the analytical approach.

**Table 3:** Results from the logistic regression of ITN household ownership on parasitemia prevalence by malaria transmission risk.

Indicator		High			Medium			Low		
		OR	*p-*Value	95% CI	OR	*p-*Value	95% CI	OR	*p-*Value	95% CI
ITN ownership		0.94	0.404	(0.81–1.09)	0.76	0.000	(0.67–0.87)	0.72	0.314	(0.38–1.37)
Seasonality	Dry	1.00	—	1.00	1.00	—	—	1.00	—	—
	Wet	1.14	0.199	(0.94–1.38)	1.89	0.000	(1.61–2.23)	3.48	0.008	(1.39–8.70)
Child's age (y)	0–1	1.00	—	1.00	1.00	—	—	1.00	—	—
	2–3	2.26	0.000	(1.91–2.68)	1.90	0.000	(1.56–2.26)	1.75	0.170	(0.79–3.90)
	4–5	2.47	0.000	(2.02–3.02)	2.34	0.000	(1.95–2.81)	2.12	0.140	(0.78–5.72)
Maternal education	None	1.00	—	1.00	1.00	—	—	1.00	—	—
	Primary	0.79	0.004	(0.67–0.92)	0.87	0.107	(0.74–1.03)	4.43	0.002	(1.77–11.1)
	≥Secondary	0.61	0.002	(0.44–0.83)	0.55	0.000	(0.39–0.76)	1.06	0.934	(0.29–3.81)
Household wealth (quintiles)	Poorest	1.00	—	1.00	1.00	—	—	1.00	—	—
	Quintile 2	1.27	0.013	(1.05–1.54)	0.75	0.001	(0.63–0.89)	0.71	0.580	(0.21–2.38)
	Quintile 3	0.82	0.073	(0.65–1.02)	0.51	0.000	(0.42–0.61)	0.69	0.511	(0.23–2.06)
	Quintile 4	0.63	0.001	(0.48–0.83)	0.50	0.000	(0.41–0.62)	0.58	0.340	(0.19–1.83)
	Richest	0.31	0.000	(0.21–0.46)	0.36	0.000	(0.26–0.49)	0.59	0.362	(0.19–1.83)
Urban residence	Rural	1.00	—	1.00	1.00	—	—	1.00	—	—
	Urban	0.70	0.002	(0.56–0.88)	0.40	0.000	(0.30–0.53)	0.46	0.196	(0.14–1.50)

**Table 4:** Results from the logistic regression of ITN use in children under five on prevalence of parasitemia by malaria transmission risk.

Indicator		High			Medium			Low		
		OR	*p-*Value	95% CI	OR	*p-*Value	95% CI	OR	*p*-Value	95% CI
ITN use		0.91	0.315	(0.77–1.09)	0.75	0.000	(0.64–0.88)	0.95	0.902	(0.45–2.02)
Seasonality	Dry	1.00	—	1.00	1.00	—	—	1.00	—	—
	Wet	1.08	0.531	(0.86–1.35)	1.93	0.000	(1.60–2.31)	4.05	0.013	(1.35–12.2)
Child's age (y)	0–1	1.00	—	1.00	1.00	—	—	1.00	—	—
	2–3	2.27	0.000	(1.86–2.76)	1.76	0.000	(1.47–2.10)	1.23	0.645	(0.51–2.95)
	4–5	2.29	0.000	(1.81–2.90)	2.46	0.000	(1.99–3.04)	1.25	0.687	(0.42–3.78)
Maternal education	None	1.00	—	1.00	1.00	—	—	1.00	—	—
	Primary	0.76	0.004	(0.62–0.91)	0.87	0.150	(0.71–1.05)	4.48	0.003	(1.66–12.0)
	≥Secondary	0.51	0.001	(0.35–0.75)	0.54	0.001	(0.37–0.79)	0.85	0.818	(0.21–3.46)
Household wealth (quintiles)	Poorest	1.00	—	1.00	1.00	—	—	1.00	—	—
	Quintile 2	1.31	0.022	(1.04–1.64)	0.82	0.053	(0.67–1.00)	0.47	0.295	(0.11–1.94)
	Quintile 3	0.80	0.097	(0.62–1.04)	0.58	0.000	(0.46–0.72)	0.61	0.384	(0.20–1.84)
	Quintile 4	0.70	0.026	(0.51–0.96)	0.56	0.000	(0.43–0.72)	0.47	0.210	(0.14–1.53)
	Richest	0.34	0.000	(0.22–0.53)	0.39	0.000	(0.27–0.57)	0.24	0.058	(0.21–3.46)
Urban residence	Urban	1.00	—	1.00	1.00	—	—	1.00	—	—
	Rural	0.67	0.003	(0.52–0.88)	0.36	0.000	(0.26–0.51)	0.39	0.187	(0.09–1.59)

**Table 5:** Results from the logistic regression of ITN household ownership on all-cause mortality among children 1 mo to 59 mo of age by malaria transmission risk.

Indicator		High			Medium			Low		
		RR	*p*-Value	95% CI	RR	*p*-Value	95% CI	RR	*p*-Value	95% CI
ITN ownership		0.82	0.001	(0.73–0.93)	0.81	0.003	(0.70–0.93)	0.74	0.094	(0.53–1.05)
Seasonality	Dry	1.00	—	—	1.00	—	—	1.00	—	—
	Wet	0.98	0.590	(0.93–1.05)	0.95	0.185	(0.88–1.02)	0.89	0.280	(0.72–1.10)
Child's sex	Male	1.00	—	—	1.00	—	—	1.00	—	—
	Female	0.98	0.463	(0.93–1.03)	0.92	0.004	(0.87–0.97)	0.94	0.358	(0.84–1.07)
Birth interval (mo)	<12	1.00	—	—	1.00	—	—	1.00	—	—
	12–23	0.84	0.150	(0.66–1.07)	0.82	0.114	(0.64–1.05)	0.85	0.526	(0.51–1.41)
	≥24	0.55	0.000	(0.44–0.70)	0.58	0.000	(0.46–0.74)	0.53	0.013	(0.32–0.87)
Birth order	First	1.00	—	—	1.00	—	—	1.00	—	—
	≥Second	0.90	0.014	(0.84–0.98)	0.94	0.184	(0.85–1.03)	0.68	0.000	(0.56–0.82)
Birth type	Single	1.00	—	—	1.00	—	—	1.00	—	—
	Multiple	2.34	0.000	(2.09–2.61)	2.23	0.000	(1.95–2.55)	2.47	0.000	(1.84–3.31)
Maternal age (y)	15–19	1.08	0.321	(0.93–1.26)	0.98	0.780	(0.82–1.16)	0.92	0.651	(0.65–1.31)
	20–24	1.07	0.128	(0.98–1.18)	0.91	0.066	(0.82–1.01)	0.69	0.001	(0.55–0.85)
	25–29	1.05	0.266	(0.97–1.13)	0.91	0.031	(0.83–0.99)	0.74	0.001	(0.62–0.89)
	30–34	1.00	—	—	1.00	—	—	1.00	—	—
	35–39	1.10	0.040	(1.00–1.20)	1.04	0.428	(0.94–1.15)	1.01	0.908	(0.83–1.24)
	40–44	0.99	0.821	(0.88–1.11)	1.10	0.144	(0.97–1.24)	1.22	0.099	(0.96–1.55)
	45–49	1.15	0.092	(0.98–1.34)	1.30	0.004	(1.09–1.55)	0.98	0.923	(0.66–1.45)
Maternal education	None	1.00	—	—	1.00	—	—	1.00	—	—
	Primary	0.95	0.177	(0.89–1.02)	0.97	0.391	(0.90–1.04)	0.84	0.033	(0.72–0.99)
	≥Second.	0.66	0.000	(0.59–0.73)	0.69	0.000	(0.61–0.78)	0.55	0.000	(0.43–0.70)
*n* household members	≤4	1.00	—	—	1.00	—	—	1.00	—	—
	5–8	0.85	0.000	(0.80–0.91)	0.75	0.000	(0.70–0.81)	0.83	0.016	(0.72–0.97)
	≥9	0.89	0.002	(0.83–0.96)	0.73	0.000	(0.67–0.80)	0.72	0.001	(0.59–0.88)
Household wealth (quintiles)	Poorest	1.00	—	—	1.00	—	—	1.00	—	—
	Quintile 2	1.01	0.698	(0.94–1.09)	1.07	0.150	(0.98–1.16)	1.01	0.917	(0.84–1.22)
	Quintile 3	1.00	0.939	(0.92–1.08)	1.00	0.972	(0.91–1.10)	1.03	0.783	(0.85–1.24)
	Quintile 4	0.95	0.312	(0.87–1.05)	1.06	0.249	(0.96–1.17)	0.92	0.404	(0.75–1.12)
	Richest	0.74	0.000	(0.65–0.85)	0.81	0.002	(0.71–0.92)	0.76	0.040	(0.59–0.99)
Urban residence	Rural	1.00	—	—	1.00	—	—	1.00	—	—
	Urban	0.91	0.017	(0.84–0.98)	1.03	0.556	(0.93–1.14)	1.17	0.135	(0.95–1.44)
PSU-SBA coverage		0.88	0.025	(0.78–0.98)	0.76	0.001	(0.65–0.89)	0.99	0.931	(0.72–1.36)
PSU-DPT3 coverage		0.70	0.000	(0.61–0.81)	0.56	0.000	(0.48–0.65)	0.69	0.021	(0.51–0.95)
Calendar year	1997	1.82	0.253	(0.65–5.09)	3.05	0.000	(2.49–3.73)	3.23	0.000	(1.82–5.71)
	1998	2.86	0.000	(1.92–4.28)	2.25	0.000	(1.89–2.69)	2.71	0.000	(1.61–4.55)
	1999	1.87	0.001	(1.31–2.65)	1.35	0.000	(1.17–1.57)	1.30	0.223	(0.85–1.99)
	2000	1.00	—	—	—	—	—	—	—	—
	2001	0.81	0.003	(0.70–0.93)	0.52	0.000	(0.44–0.62)	0.89	0.534	(0.62–1.28)
	2002	0.83	0.009	(0.72–0.95)	0.35	0.000	(0.29–0.42)	0.64	0.017	(0.45–0.92)
	2003	0.78	0.002	(0.66–0.91)	0.38	0.000	(0.31–0.46)	0.66	0.032	(0.45–0.97)
	2004	0.75	0.002	(0.62–0.90)	0.32	0.000	(0.26–0.40)	0.64	0.025	(0.44–0.95)
	2005	0.72	0.001	(0.60–0.88)	0.30	0.000	(0.24–0.37)	0.66	0.056	(0.44–1.01)
	2006	0.72	0.001	(0.59–0.88)	0.29	0.000	(0.22–0.37)	0.58	0.027	(0.35–0.94)
	2007	0.75	0.009	(0.60–0.93)	0.30	0.000	(0.22–0.39)	0.75	0.308	(0.43–1.30)
	2008	0.69	0.003	(0.54–0.88)	0.27	0.000	(0.19–0.37)	0.56	0.156	(0.25–1.25)

Child age in months was included as analysis time. Both calendar year and seasonality were allowed to vary analysis time.

DPT3, three-dose diphtheria, pertussis and tetanus.

**Figure 3:**
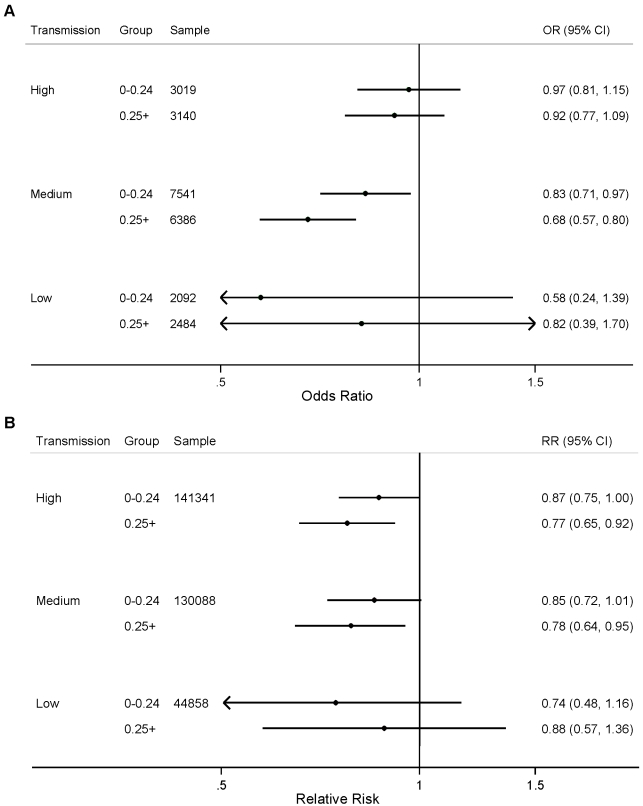
Effect of ITNs on (A) prevalence of parasitemia; and (B) all-cause mortality among children 1 mo to 59 mo of age, stratified by number of ITNs per household member (<0.25 ITNs per household member, ≥0.25 ITNs per household member) and malaria transmission risk (high, medium, low).

**Figure 4:**
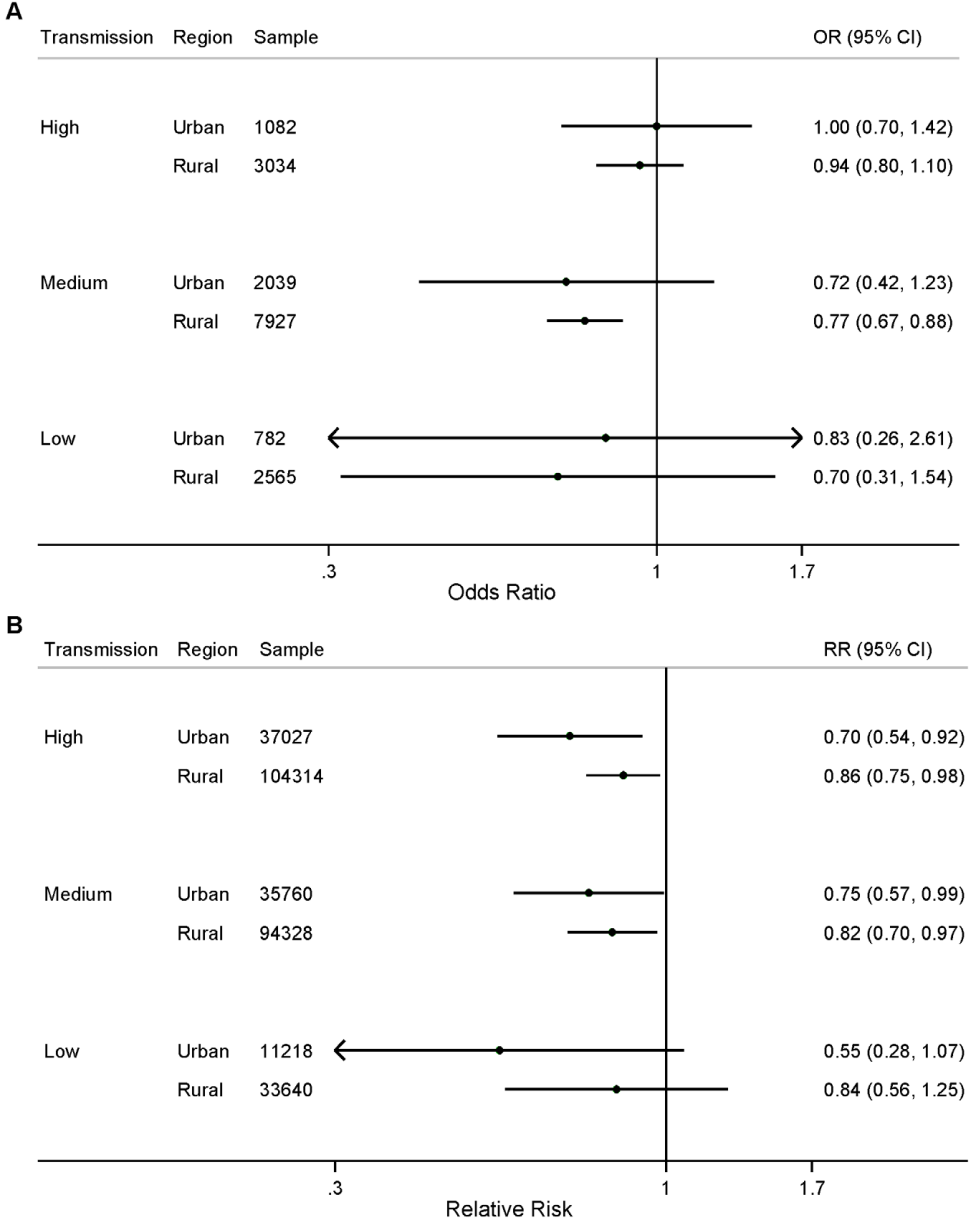
Effect of ITN ownership on (A) prevalence of parasitemia; and (B) all-cause mortality among children 1 mo to 59 mo of age, stratified by area of residence (urban or rural) and malaria transmission risk (high, medium, low).

We did not observe statistically significant differences in the effect of the number of ITNs per household member for either parasitemia prevalence or child mortality when stratified by transmission level (Figure 3; *p*>0.05). We found a statistically significant association between ITNs and child mortality in urban areas with high and medium levels of malaria transmission (Figure 4); however, we did not observe statistically significant differences in the effect of ITNs in rural versus urban areas when stratified by transmission level (Figure 4; *p*>0.05).

## Discussion

Our findings from a large number of countries suggest that the rapid scale-up in ITN coverage observed in several sub-Saharan African countries has likely been accompanied by reductions in child mortality. Our results are also highly consistent with findings from previous RCTs. We found a 23% (95% CI 13%–31%) pooled relative reduction in child mortality across 29 surveys compared to the pooled 18% (95% CI 10%–25%) relative reduction observed in three RCTs [3]. For parasitemia, we found a 20% (3%–35%) reduction across seven surveys, which is not statistically distinguishable from the pooled 13% reduction observed in seven RCTs [4]. The lack of a major difference between the RCTs and our analysis may be partly explained by the intention to treat analysis used in RCTs, although ITN coverage in the RCTs was almost universal. It is also important to note that the RCTs targeted provision of ITNs across all age groups, while the scale-up in most sub-Saharan African countries has initially focused on children and pregnant women.

Our results are also consistent and statistically indistinguishable from previous observational studies of ITNs on child mortality. A cohort study in Kenya found a 44% (4%–67%) relative reduction in mortality among children age 1 mo to 59 mo associated with ITN use [8]. A case-control study in Tanzania found a 27% (95% CI 3%–45%) relative reduction in mortality among children aged 1 mo to 4 y associated with ITN use [11]. Our analysis adds to the existing literature by providing evidence of the effect of ITNs on health outcomes under routine conditions over a much broader range of transmission levels and countries; previous studies were predominantly in high endemicity areas. Overall, this finding suggests that on average at least, ITNs have a similar and sizeable effect on health outcomes under routine use compared to that seen in efficacy trials.

This finding supports the continued scale-up of ITNs in sub-Saharan Africa, such as the more recent efforts in Nigeria and Democratic Republic of Congo that had previously low levels of ITN coverage and large populations at risk of malaria [1]. It also emphasizes the importance of ongoing and future efforts to maintain coverage of ITNs in those countries with successful scale-ups by replacing worn out ITNs. Furthermore, it also suggests that the massive effort to scale up ITN coverage over the past decade has paid off and that it is possible for health systems to increase coverage of interventions and affect health outcomes over a relatively short period of time. Continued coordinated efforts between local and national governments, international organizations, funding agencies, and researchers are needed to ensure that ITNs are reaching all populations at risk of malaria. With the relatively large impact of ITNs on child mortality, our findings also support the continued emphasis on malaria control more generally, including the push towards malaria elimination, as a way of improving child health in endemic countries.

We found no evidence of substantial heterogeneity in the effect of ITNs on child mortality across the countries studied here. On the other hand, we found evidence of heterogeneity in the association between ITNs and parasitemia prevalence across countries. One possible explanation is because parasitemia may persist for some time after initial malaria infection; this heterogeneity may reflect different levels of malaria transmission intensity. That is, in high transmission areas, parasitemia may be so prevalent that it is a poor indicator of the incidence of malaria. This heterogeneity in the effect of ITNs on parasitemia prevalence is an important topic for future investigation. We were also not able to detect a significantly different effect on parasitemia of children sleeping under an ITN compared to just household ownership of an ITN; this may simply reflect limited statistical power to detect a true difference. However, we must examine other possible explanations. One possible explanation is that even though MIS data collection is designed to be in high transmission seasons, some of the data collection does occur in low transmission seasons and as the MIS only record information about sleeping under an ITN for the previous night, use of ITNs by children during the low transmission season may not be indicative of use in high transmission seasons. Mothers responding to a question by interviewers about whether their child slept under an ITN the previous night may also be more likely to respond in the positive because of social pressure.

In our study we were not able to detect significant differences in the effect of ITNs by transmission level, number of ITNs owned per household member, or urban and rural residence. These findings likely reflect inadequate power, as indicated by the width of the confidence intervals, to detect statistically significant differences. A previous meta-analysis of RCTs suggested that the efficacy of ITNs is lower in areas with higher malaria transmission [4], while an observational study from rural Kenya [8] found greater effects in areas of high malaria transmission. In our pooled analysis we found significant effects of ITNs in urban areas, which supports previous studies that have shown significant impacts of ITNs on malaria outcomes in urban areas [27],[28]. We were also able to detect significant impacts of ITNs in only a limited number of individual surveys because of small sample sizes, and in general, we did not have the power to detect significant differences between surveys. On the basis of our analysis we cannot discount the possibility that the effect of ITNs varies by these and other factors, such as the extent of education on the proper use of ITNs that are accompanied with distribution programs. Given the large investments in malaria control over the past 10 y, future research and better ways to monitor how the impact of malaria control interventions might vary across populations are required.

Our study provides a method for understanding the real-world impact of not only ITNs but also other interventions on health outcomes using data that are routinely collected. There are, however, a number of limitations of our analysis. First, several MIS do not specify whether the parasitemia tests were based on microscopy or rapid diagnostic test (RDT), and as a result we were not able to standardize the parasitemia measurements. Second, our analysis was limited to publically available datasets; therefore we were not able to access the full range of MIS that have been conducted, although steps are being taken to make these data more widely accessible (e.g., www.malariasurveys.org). Third, in our analysis of parasitemia, we were limited to a cross-sectional analysis and were therefore not able to determine whether ITN exposure occurred prior to malaria infection. Fourth, we were only able to examine the relationship between ITNs and all-cause mortality as the surveys we used do not include information on cause-specific mortality. Increased use of verbal autopsy may allow for refined assessment of the impact of ITNs on malaria-specific mortality, although concerns have been raised about the predictive power of verbal autopsy for malaria [29]. Sixth, the DHS do not collect information on skilled birth attendance and immunizations for children who have died, so in our analysis we could only control for use of these interventions at the PSU level. Seventh, we were not able to control for the effect of other malaria interventions such as indoor residual spraying or drug treatment. Finally, our analysis, like others based on observational studies, may be prone to residual confounding that has not been controlled for by the methods used.

Monitoring and evaluation of interventions to improve population health must include not only measurement of utilization but also whether the delivery of the intervention at scale results in real-world changes in health outcomes. The latter is critical if we are to understand whether interventions are being delivered and used correctly. We used routinely collected survey data to assess the association between intervention use and health outcomes across a large number of countries. Our results suggest that, on average, the scale-up of ITNs in sub-Saharan Africa has led to significant reductions in child mortality—comparable to those found in previous RCTs. While further work is needed to elucidate possible variations in the effect of ITNs, these findings add to the body of evidence that ITNs are effective under usual program conditions and support the continued efforts to scale-up ITN coverage in sub-Saharan Africa.

## Abbreviations

CI: confidence interval
DHS: Demographic and Health Surveys
ITN: insecticide-treated mosquito net
LLIN: long-lasting insecticide-treated mosquito net
MIS: Malaria Indicator Surveys
OR: odds ratio
PSU: primary sampling unit
RCT: randomized controlled trial
RR: relative risk
